# Assessment of Validated Instruments for Measuring Cooking Skills in Adults: A Scoping Review

**DOI:** 10.3390/foods13233933

**Published:** 2024-12-05

**Authors:** Maísa Fernandes Caixeta Lins, Raquel Braz Assunção Botelho, Bernardo Romão, Maria Luiza Torres, Nathalia Sernizon Guimarães, Renata Puppin Zandonadi

**Affiliations:** 1 Graduate Program in Human Nutrition, Department of Nutrition, University of Brasília, Brasília 70910-900, Brazil; 2 Department of Nutrition, University of Brasília, Brasília 70910-900, Brazil; raquelbotelho@unb.br (R.B.A.B.); bernardolima156@gmail.com (B.R.); 3 Departament of Nutrition, Centro Universitário de Brasília—UniCEUB, SEPN 707/909, Brasília 70790-075, Brazil; nutri@malutorres.com; 4 Departament of Nutrition, Nursing School, Federal University of Minas Gerais, Belo Horizonte 30130-100, Brazil; nasernizon@gmail.com

**Keywords:** cooking skills, cooking skills questionnaires, food skills evaluation, healthy diet, instruments

## Abstract

Home-cooked meals are linked to healthier diets, but assessing cooking skills accurately remains challenging. This review aimed to evaluate and compare available validated instruments to assess adult cooking skills; the Joanna Briggs Institute’s manual and PRISMA-ScR checklist were followed. A search was conducted in April 2024 in five databases using MeSH Terms and adaptations, including studies written without time or language restrictions and with validated instruments to assess adult cooking skills. Reviews, conference abstracts, books, chapters, and case reports were excluded; 1070 studies were identified, and 38 remained after removing duplicates and applying eligibility criteria. Eleven different instruments were identified. Some instruments have been successfully adapted and validated in various countries, covering a broad range of skills, such as meal organization, preparation and cooking techniques, providing a thorough assessment of cooking skills. The Cooking and Food Skill Confidence Questionnaire was considered the best available model, considering its items and domains, direct relation to cooking skills, and easy translation into other cultures. The analysis revealed significant variations in the scales used, with some instruments offering detailed assessments of specific cooking techniques and easy cultural adaptation while others focused more on confidence and attitudes.

## 1. Introduction

Consuming food prepared at home has been related to healthier and more adequate diets, and knowledge regarding cooking and meal production allows better food choices, helping to prevent various public health problems such as chronic diseases and non-communicable diseases and their risk factors [1,2,3,4]. However, it is a fact that the population has increased the consumption of practical and ready-to-eat meals prepared by the food industry and food services, mainly bought or consumed out of home [5].

It is also known that these kinds of meals tend to have many calories, refined carbohydrates, saturated fatty acids (SFAs), and salt [2,6]. On the other hand, the isolated fact of eating at home does not imply healthy consumption since it depends on the individual’s social status, culture, purchasing power, availability of time to plan and prepare meals, individual perception of food, health, cooking habits, knowledge, and skills [7,8,9].

Cooking skills can be evaluated using several indicators, and sometimes, evaluating cooking skills is confused with the evaluation of how often meals are prepared and whether they are wholly or partially prepared at home [10]. However, parameters directly related to cooking skills are still a gap in the literature [11].

Several studies show the importance of assessing cooking skills, and there are several instruments around the world to assess cooking skills [2,7,11,12,13,14,15]. Previous reviews aimed to examine the assessment of personal cooking and food skills and evaluate the psychometric properties of instruments measuring those abilities [16,17].

However, none of them compared the instruments to find those that offer more accurate information about cooking skills, the domains of the instruments, and the strengths and limitations of each instrument. Also, considering the increased number of recent publications on this topic, it is crucial to identify and analyze the instruments available worldwide to evaluate cooking skills.

Although cooking skills are recognized as important to achieving a healthy diet, it is necessary to find instruments that offer accurate and complete information about these skills. This would enable further research to explore the connection between cooking skills and health. Therefore, this scoping review sought to analyze and compare available validated instruments for assessing adult cooking and food skills.

## 2. Materials and Methods

This scoping review followed the Joanna Briggs Institute criterion. The protocol was structured following Peters et al. [18] and Arksey and O’Malley [19], and enhanced by Levac et al.’s [20] recommendations. It was prepared according to the Preferred Reporting Items for Systematic Reviews and Meta-Analyses Extension for Scoping Reviews (PRISMA-ScR) checklist.

This scoping review followed the steps below: (1) establish the research question; (2) find pertinent studies; (3) select studies; (4) chart the data; (5) group, summarize, and describe the results. The scoping review protocol was recorded on Open Science Framework to verify ongoing reviews and avoid unnecessary duplication of research under site <osf.io/g86wd>.

To enhance the organization of the research question and the eligibility criteria, we used the mnemonic PCC (Population, Concept, Context), described by the research question: P—Adults; C—Cooking Skills; C—Available Validated Instruments.

## 3. Results

**Figure 1:**
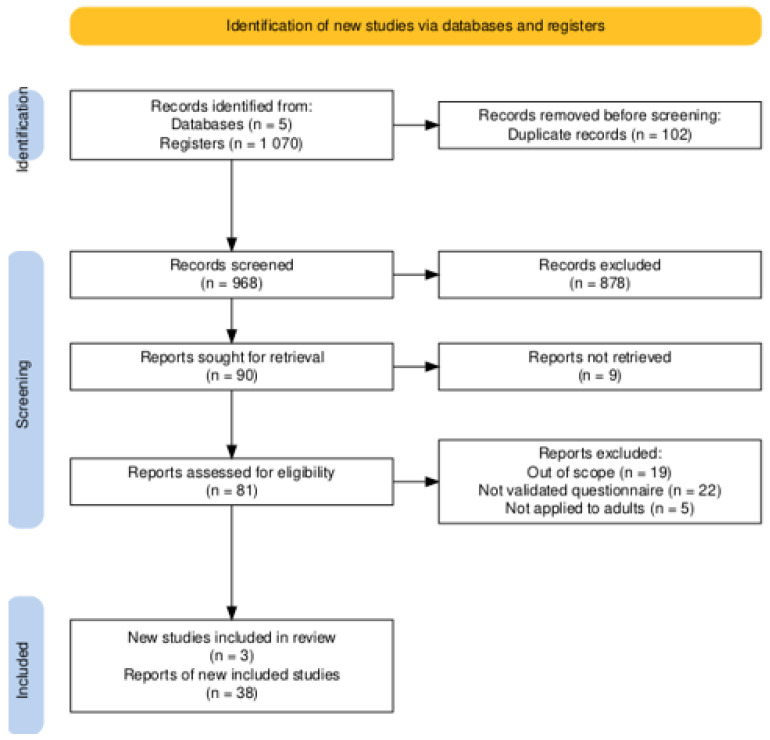
Flowchart of the literature search and selection process, adapted from the PRISMA guidelines.

Following a systematic literature search and analysis, 1.070 studies were found in databases. After duplicate exclusion (n = 102), an analysis of 968 studies’ titles and abstracts was conducted, and only 81 of them were chosen for full-text reading, considering the inclusion and exclusion criteria (Figure 1). By reading full texts, 19 studies were considered outside the scope, five were excluded as they targeted children and adolescents, and 21 were excluded for not utilizing a validated instrument, resulting in a total exclusion of 45 articles at this stage. Subsequently, three studies were added following a thorough verification of the references of the 36 studies. Therefore, 38 studies released from 2006 to 2024 were incorporated into this review. The process of study identification and selection is outlined in the flowchart presented in Figure 1.

### 3.1. Studies’ General Characteristics

**Figure 2:**
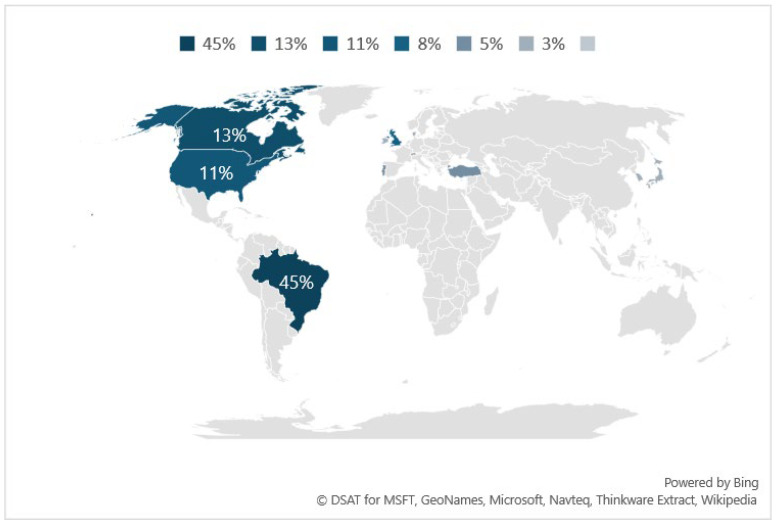
Studies published around the world distribution included in this review.

The studies using 11 validated instruments for measuring cooking skills were performed in 10 different countries (Figure 2). Most of them were conducted in Brazil (n = 17; 45%), followed by Canada (n = 5; 13%), United States of America (n = 4; 11%), United Kingdon (n = 3; 8%), and Portugal (n = 2; 5%). Denmark, Ireland, Japan, South Korea, and Switzerland (n = 1; 3%) presented only one study each (Figure 2).

A total of twelve distinct instruments were utilized across the studies to assess cooking skills: (i) Food and Cooking Skills Questionnaire; (ii) Short questionnaire for assessing the impact of cooking skills interventions; (iii) Cooking Skills Scale; (iv) Canadian Community Health Survey (CCHS) Rapid Response Annex on Food Skills; (v) Cooking and Food Skill Confidence Questionnaire; (vi) Cooking Skills Index (CSI); (vii) Cooking and Food Provisioning Action Scale (CAFPAS); (viii) Scale to Measure Cooking Skills; (ix) Food Skills Questionnaire (FSQ); (x) Japanese Cooking Skills Scale used in the Japan Gerontological Evaluation Study (JAGES); (xi) Escala de Habilidades Culinárias Domésticas da Atenção Primária à Saúde (EHAPS).

Table 1 presents the instruments and the respective studies using them, including the authors, year of publication, study objective, form of application, and whether the translated or original version was used. The characteristics of each instrument are described in the specific topic of this review.

Table 2 provides a concise overview of each instrument, its domains, and the description of the items grouped within each domain for better visualization.

**Figure 3:**
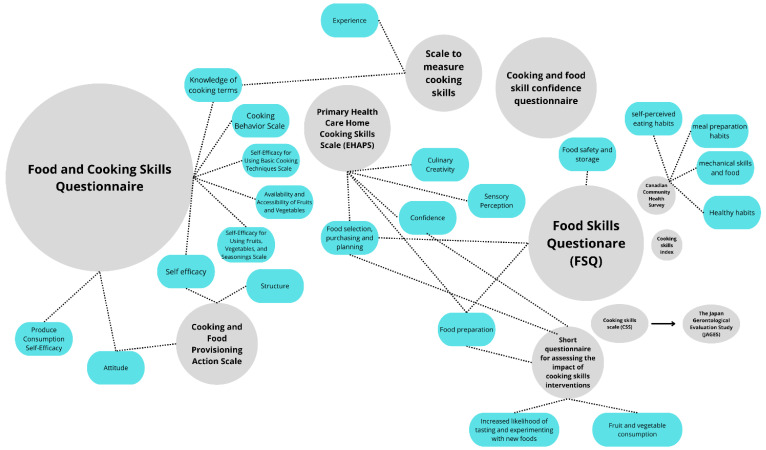
Instruments for evaluating cooking skills and their domains. The circles’ sizes represent the number of studies the instrument was applied to.

The instruments presented 22 different domains (Table 2). Some of them were common for more than one instrument (Figure 3). The Food and Cooking Skills Questionnaire is the one that presents the largest number of domains (n = 8), followed by Food Preparation Knowledge (n = 7), Escala de Habilidades Culinárias Domésticas da Atenção Primária à Saúde (EHAPS) (n = 5).

Two domains are used in three instruments: (i) food selection, purchasing and planning, and (ii) food preparation. Four domains are used in two instruments: (i) self-efficacy; (ii) knowledge of cooking terms; (iii) food safety and storage; and (iv) attitude (Figure 3). It is essential to observe that similar denominations of the domains in different instruments may present different constructs depending on the instrument, and domains with different denominations may evaluate similar constructs.

In Figure 3, the instruments presented in this review and their respective domains are represented. Each gray circle represents an instrument, and its size indicates the number of published articles using that instrument. The blue circles represent their respective domains, as well as the similarity of these domains across different instruments used worldwide.

As observed in Figure 3, not all instruments utilized domains in their composition. The presence or absence of domains is simply a way to stratify and better organize the instrument for full comprehension and to facilitate interpretation. However, the absence of a domain does not imply a less effective instrument; sometimes, instruments without domains are more concise and to the point. This review observed that even with different nomenclatures, some domains were common among the various instruments.

### 3.2. Characteristics of the Instruments Used to Evaluate Cooking Skills

#### 3.2.1. The Food and Cooking Skills Questionnaire

This was the most used instrument to evaluate cooking skills, applied in 16 studies (41%) in three different countries (USA, UK, and Brazil) with two distinct and validated language versions (Table 1).

Its original English version was used in one study in the USA and another in the UK. In Brazil, the instrument was translated and validated into Brazilian Portuguese [25] and employed in many studies (n = 16; 37.3%), showing its widespread utilization in this country [55].

Considering that Brazil has the largest number of studies (Figure 2) and this is the most used questionnaire in Brazil, it is important to highlight that this questionnaire was the first instrument translated and validated into Brazilian Portuguese, first published in this country in 2017 (ten years after the original version) by the same research group in all the studies.

This instrument is composed of eight domains, with a total of 63 items (Table 2). It is a lengthy questionnaire that, besides cooking skills, evaluates attitudes, behaviors, and self-efficacy among participants. It employs various scales in those eight different domains.

The Availability and Accessibility of Fruits and Vegetables (AAFV) domain uses a simple yes/no response format, where participants indicate the presence or absence of these foods in their environment, providing a straightforward measure of their practical access to essential dietary components. In the Cooking Attitude (CA) domain, a five-point Likert scale varying from “strongly disagree” to “strongly agree” is employed to evaluate participants’ feelings regarding cooking, including their enjoyment and perceptions of its healthiness and affordability. Some items in this domain are reverse-coded.

The Cooking Behavior (CB) domain uses a frequency-based scale to evaluate how often participants engage in various cooking activities, ranging from “not at all” to “about every day,” thus measuring their cooking practices.

Self-efficacy is assessed across several domains, each using a five-point Likert scale varying from “not at all confident” to “extremely confident.” The Self-Efficacy in Produce Consumption (SEPC) domain measures participants’ confidence in meeting daily fruit and vegetable intake recommendations. In contrast, the Cooking Self-Efficacy (SEC) domain evaluates their confidence in preparing basic meals. Similarly, the Self-Efficacy for Using Cooking Techniques (SECT) domain assesses confidence in specific cooking techniques, and the domain Self-Efficacy for Using Fruits, Vegetables, and Seasonings (SEFVS) gauges capability in incorporating fruits, vegetables and seasonings into meals.

In contrast, the Knowledge on Cooking Terms and Techniques (CTT) domain uses a multiple-choice format to objectively assess participants’ understanding of cooking terms and techniques, providing a measure of their foundational cooking knowledge, which underpins their practical skills.

This last domain had an interesting proposal on the original version of the instrument. It helped to compare the other domains’ results to guide the discussion about the individual’s confidence. For the CTT measure, participants who successfully answered to at least 75% of the domain’s items (6 items or more) were categorized as high level of knowledge, and those who achieved ≤60% (≤5 items) were classified as low level of knowledge.

Therefore, the other domains’ results were calculated using the mean level of responses on the presented scale (Table 2), and this result was compared with the individual’s level of knowledge assessed in the CTT. For example, supposing the person’s average on the Cooking Attitude Scale was higher when the CTT was also high, they would say that individuals with more knowledge have a higher average on the Cooking Attitude Scale.

The same approach was used in translations. The most used version of this instrument in Brazilian Portuguese employed, sometimes, the same parameter in its analyses; in other cases, they only compared the average before and after an intervention, but always used the average per domain for discussion.

In this Brazilian Portuguese version, they proposed an evaluation of the instrument defining the individuals with scores between 20 and 44 as having low cooking skills, the ones that obtained between 44 and 73 as having medium cooking skills, and the respondents with more than 73 going up to 100 points as having high cooking skills. This addition was made in each domain of the instrument, also giving an interval to define the person as high, medium or low in each domain. As scores are derived from answers on a Likert scale, this was calculated by summing the number of answers the person made from 1 to 5. In this way, a domain with 8 items has a minimum possible score of eight and a maximum of 40 points.

Since this study aims to discuss the instruments available for assessing cooking skills, it is crucial to emphasize that among all these domains, only the “Knowledge of Cooking Terms and Techniques evaluation” (r = 0.75), “Cooking Self-Efficacy (SEC)” (r = 0.89) and Self-Efficacy for Using Cooking Techniques (SECT) (r = 0.89) seem to evaluate participants’ understanding of cooking skills and demonstrate good reliability.

#### 3.2.2. Cooking and Food Skill Confidence Questionnaire

The second most used instrument was “Cooking and food skill confidence questionnaire” which originally was in English and was applied in six different studies. Originally developed and published in 2017 [11], the questionnaire was validated into multiple languages, including Turkish [43], European Portuguese [41], and Brazilian Portuguese [44]. These translations highlight this instrument’s practicality and cultural adaptability, showing its potential for use in more countries.

A significant positive relationship was identified between the domain of cooking and food skill confidence domains (r = 0.76, *p* < 0.001), indicating closely associated components. To validate the original instrument, the authors applied it to two groups, one considered to have high skills and the other low skills. As a result, the measures demonstrated strong convergent validity due to their close association. Furthermore, their ability to distinguish between individuals with high and low skills highlighted their robust discriminant validity.

The first domain, “Cooking Skills Confidence Measure”, comprises 14 items that evaluate participants’ competence in various cooking methods and food preparation techniques. These items include fundamental cooking tasks such as chopping, blending, steaming, boiling, roasting, frying, baking, and preparing raw meat, poultry, and fish, as shown in Table 2.

It also assesses the ability to make sauces from scratch and to use herbs and spices for flavoring. Participants rate their level of confidence in performing each task on a scale from 1 (very poor) to 7 (very good), with an additional option to indicate if they “never/rarely do it.” This scale is crucial for understanding the practical cooking abilities of individuals and their confidence in performing essential cooking tasks, which are vital for preparing meals at home.

The “Food Skills Confidence Measure”, on the other hand, includes 18 items that cover a broader range of skills necessary for effective meal preparation and management. For instance, the measure assesses confidence in planning ahead for meals, making purchases using a grocery list, evaluating prices before buying, and using leftovers to prepare new dishes.

It also evaluates participants’ ability to prepare meals with limited ingredients or time and to read and interpret food labels, such as best-before dates and nutritional information. Like the cooking skills measure, participants rate their confidence in each task on a seven-point Likert scale. This comprehensive evaluation of food skills is essential for understanding how individuals manage food resources and plan and prepare meals, which can significantly impact their cooking skills. All the translations use these same scales and items.

This questionnaire provides a robust assessment of cooking proficiency and practices. Its concise set of items, minimal cultural dependence, and direct evaluations of essential home cooking skills and behaviors make it ideal for various settings. All the translations of this questionnaire have been validated, and for all of them, similar results have been confirmed for internal consistency, demonstrating the validity of this instrument.

#### 3.2.3. Short Questionnaire for Assessing the Impact of Cooking Skills Interventions

The Short questionnaire for assessing the impact of cooking skills interventions is a quick instrument designed to be completed in less than 10 min and with few items. They performed the instrument’s validation in four phases: Content validity, Face validity, Reliability testing, and Feasibility testing; all of them received satisfactory results in statistical analysis. The instrument assesses the individual’s behaviors and routine in food preparation, but no question is directed at cooking skills. No question in this instrument measures the person’s skills in peeling, boiling, or frying something.

Table 2 shows each item in each domain of this instrument. There are five domains but these are not clearly divided into items; this is why, in Table 2, they are not explicit. One domain is called Confidence in using a recipe where the person must answer the kind of recipes or type of meals they are cooking at the present and their abilities of food preparation. The other is to identify the frequency with which the person uses basic ingredients for preparing meals, and the third domain is about buying less convenience food to identify the individual’s food selection, purchasing and planning.

The fourth domain focuses on the greater tendency to try and experiment with unfamiliar foods, which includes items related to sampling foods they have not eaten before and preparing and cooking new foods and recipes. The last one is about fruit and vegetable consumption, indicating how frequently they consume different foods, including fruits, vegetables, pasta, rice, and fish. All those domains do not directly evaluate cooking skills. In addition, this instrument uses different scales (multiple-choice, 7-point Likert, and total items) that can make the interpretation of the results difficult.

#### 3.2.4. Cooking Skill Scale (CSS)

The cooking skill scale (CSS) instrument is very synthesized, with only seven items; it is aimed at the individual’s self-perception of cooking skills. The questionnaire does not include questions about more nuanced cooking techniques, such as knife skills, timing, seasoning, or the ability to adjust recipes based on available ingredients.

CSS was also translated, adapted, and validated into European Portuguese [37] and applied using the same scale in a straightforward translation of the questions, demonstrating a particular quality of replicating this article in another country. This version was validated and demonstrated a high Cronbach’s α of 0.90, indicating strong internal consistency. Test–retest analysis revealed consistent mean scores between administrations, with Spearman’s rank correlation coefficient at 0.79, reflecting good repeatability. Besides the translation version in European Portuguese, this instrument was translated and adapted, composing a new instrument in Japan [56].

The instrument includes seven items that assess various aspects of cooking ability (Table 2). Respondents rate their skills on a six-point scale varying from 1 (“Do not agree at all”) to 6 (“Totally agree”). The items evaluate self-perceived cooking sufficiency and specific cooking abilities, such as preparing a meal without a recipe, making dishes like gratin, soup, sauce, baking cake and bread. The European Portuguese version used the same items and scale. This instrument was translated into Japanese, resulting in another instrument based on CSS that was used in The Japan Gerontological Evaluation (JAGES) [53]; however, the authors need to adapt and validate it.

Although this is a short instrument and does not directly assess cooking skills, it could be developed further to include more items for such analysis. However, it is important to remember the complexity involved in translating this instrument, which may require creating a new version. Therefore, evaluating various available options is essential to decide which is the best instrument to apply worldwide.

##### Cooking Skills Scale Adaptation—The Japan Gerontological Evaluation Study (JAGES)

The Japan Gerontological Evaluation Study (JAGES) used the modified version of the CSS as part of a national project in Japan to evaluate the aging population [56]. The Japanese version adapted from CSS regarding cooking skills was applied to assess the relationship between cooking skills and Japanese individuals’ quality of life. It was the only one made with an aging population, as all the others cited in this review were validated and tested/applied with adults.

The process included reliability testing through internal consistency measures, where Cronbach’s alpha was calculated to ensure the coherence of the items within the scale, showing a good reliability (0.84). Additionally, construct validity was assessed using factor analysis [53], revealing a clear factor structure that aligned to theoretical framework of cooking skills [53].

The number of items and the scale used in this version was the same as the original one, as described in this section and shown in Table 2. For the cultural adaptation, all the items were modified to be more specific for the Japanese population’s practices. The fact that it has undergone so many changes may even indicate a certain fragility in this instrument, even though adaptations are sometimes necessary. Since the questions address very specific techniques or preparations, changes in culture, region, or population habits may directly affect the results obtained by this instrument.

#### 3.2.5. Canadian Community Health Survey—Cooking Skills Section

The Canadian Community Health Survey—cooking skills section was used in three studies; two were applied in Canada [38,39], and one in Norway [56]. However, when applied in Norway, the authors only translated the questions for evaluating cooking skills into Norwegian, Somali and Arabic languages without validating these translations, so the study was not included in this review.

It is part of a nationwide population analysis survey, and no statistical analysis was conducted for its validity. This instrument was developed by Statistics Canada in 2013, and its results were published in 2016 in the same country [38].

As described in Table 2, the domain assessing self-perceived eating habits consists of a single question where respondents rate their eating habits as “Excellent or Very Good”, “Good or Fair”, or “Poor”. The domain focused on meal preparation habits includes five questions examining the foods most frequently used in meal preparation.

The domain on mechanical skills and food conceptualization is more extensive, containing eight questions that cover specific food preparation tasks, including using a knife, peeling and chopping vegetables, freezing vegetables, canning from raw ingredients, and cooking raw meat, poultry, or fish, with respondents rating their proficiency in each task.

The domain assessing cooking skills comprises two questions: one that evaluates basic cooking skills, such as the ability to prepare simple meals like cooking an egg or preparing a grilled cheese sandwich, and another that assesses more advanced cooking skills, such as preparing most dishes, particularly when following a recipe.

The domain related to healthy habits questions whether the participant does or does not make modifications in the recipes to make it healthier or if they grow any herbs at home, for example. It is a particularly individual perception of health, which can make it difficult to replicate this instrument worldwide.

Although this instrument includes questions specifically targeting cooking skills, such as cutting, peeling, and boiling, it is more challenging to replicate because it has questions too specific in some cultures, like baking a muffin (instead of just baking) or cooking a soup or casserole. This may explain why it has not been validated for use internationally. This instrument was only applied in Canada in a validated way, which enforces this idea.

#### 3.2.6. Cooking and Food Provisioning Action Scale (CAFPAS)

A positive aspect of Cooking and Food Provisioning Action Scale (CAFPAS) is that this original instrument provides a methodology for calculating a score to measure an individual’s cooking skills, as addressed in another section of this review. The validation was carried out, and the scales exceeded >0.70 in all cases, demonstrating excellent internal consistency.

To assess the effectiveness of the summary CAFPAS score, its correlation with a single-factor principal component analysis (PCA) of the Self-Efficacy, Attitude, and Structure scores (derived through regression from the EFA solution) was analyzed across the entire sample. The first principal component explained 62% of the variance in the three scales, and the correlation between PC1 and CAFPAS was exceptionally strong (=0.99, 95% CI: −0.99 to 1.000, *p* < 0.05). The internal remarkably strong (0.91 for Self-Efficacy, 0.86 for Attitude, and 0.81 for Structure).

The Cooking and Food Provisioning Action Scale (CAFPAS) comprises 28 items administered using a 7-point bipolar Likert scale. To calculate subscale scores for an individual, each response option is coded from 1 to 7, with some items reverse-scored as needed (see Table 2). The total score for each subscale is then obtained by summing all the items within that subscale and dividing by the standard deviation of the sample population’s scores on the subscale. An overall “CAFPAS Score” can be calculated by summing the individual subscales. For example, no parameter was established in the study to classify the score as low, medium, or high.

When translated into Korean, the translation and back-translation method was followed, adhering to the suggested standard for this methodology [48,57]. The internal consistency of the translated questionnaire resulted in a Cronbach’s alpha very similar to the original (0.83), indicating that both versions exhibited consistency and reinforced the quality of this instrument’s reproducibility in different versions.

This application of the instrument in another country, with a different culture, and validated statistically, reinforces its potential for replication without the necessity of major adaptations that modify the instrument such as in the Japanese version of Cooking Skills Scale mentioned in Section Cooking Skills Scale Adaptation—The Japan Gerontological Evaluation Study (JAGES). However, the questions used in this instrument are heavily related to attitude, self-perception of capability in food preparation, and even routine and available time to prepare recipes or meals. None of the questions directly assess cooking skills such as peeling, cutting, baking, or cooking the food, as shown in Table 2.

#### 3.2.7. Scale to Measure Cooking Skills

The Scale to Measure Cooking Skills includes items highly focused on cooking skills, making it quite relevant to the theme of this review. However, it assesses how often an individual uses a particular technique, but not necessarily whether they consider themselves proficient in that skill. Additionally, the second group of questions uses true-or-false response options, and it is more related to the individual’s knowledge of good food handling practices rather than the skills themselves, somewhat deviating from the core aspect of the evaluation.

This scale is part of a study composed of other scales that are not related to cooking skills. In statistics analysis, it was found that all six scales (consisting of shortlisted items) are cumulative; in the validation process, no significant differences were found in *t*-tests analysis (*p* < 0.05). Additionally, an analysis of the cross-loadings reveals highly robust correlations of all items and their designated constructs, thereby confirming discriminant validity.

In the first domain, they measure the cooking experience of the participant; each item in this group is answered with a simple “Yes” or “No” to indicate whether the respondent has prepared the dish or performed the task within the last year (Table 2). It includes 21 different types of food or dishes, such as “carrot slaw,” “sauce béarnaise,” “mussels,” and “and garlic.” The second group assesses respondents’ knowledge of essential food safety practices and related information, where they are required to answer these items as “True” or “False”. This part of the scale measures their understanding of essential food safety principles, contributing to their overall cooking competence score.

This comprehensive scale effectively captures both practical cooking skills and theoretical knowledge of food safety, providing a robust measure of overall cooking competence. The cumulative approach in the first domain allows for differentiation among respondents based on the complexity of tasks they can perform. In contrast, the second domain ensures that respondents possess the necessary knowledge to handle food safely.

Additionally, as mentioned before, the questions in the first group mention very specific preparations that can be difficult to apply to other populations, as in the Cooking skill scale (CSS) adaptation to Japanese (Section 3.2.4 and Section Cooking Skills Scale Adaptation—The Japan Gerontological Evaluation Study (JAGES)). This could be why this instrument was never translated into any other language.

#### 3.2.8. Food Skills Questionnaire (FSQ)

The Food Skills Questionnaire (FSQ) effectively analyzes cooking skills through a combination of factors, which can be very efficient. This instrument was validated in different phases: Content validity, Face validity, and Reliability. In all of them, the statistical analysis results were satisfactory.

What differentiates it is that many other questions are addressed regarding the frequency of certain practices, such as checking expiration dates, and meal planning, and questions related to good practices like washing hands and washing fruits and vegetables, among others. These additional questions make the questionnaire lengthy and deviate from the central theme (evaluation of cooking skills). As mentioned in Table 2, it is structured into three primary domains: Food Selection and Planning, Food Preparation, and Food Safety and Storage.

The domain of Food Selection and Planning comprises nine items that assess respondents’ abilities in areas such as budgeting for groceries, meal planning, and selecting fruits and vegetables, with most questions utilizing an 11-point scale varying from 0 (not confident at all) to 10 (extremely confident) to measure confidence or frequency of performing these tasks.

The Food Preparation domain, which is the largest, includes twenty items that evaluate practical cooking skills, including preparing meals from basic ingredients, following recipes, and using various cooking techniques like boiling, frying, or baking. The first four questions in this domain are composed of a multiple-choice question, followed by five questions where the person must answer from totally disagree to totally agree. The last 12 items are rated on an 11-point scale from 0 (not confident at all) to 10 (extremely confident). Lastly, the Food Safety and Storage domain consists of ten items that measure respondents’ knowledge and practices related to food safety, such as checking best-before dates, washing hands before preparing food, and ensuring food is cooked to the correct internal temperature. The questions in this domain primarily use a 5-point scale ranging from “Never” to “Always.” The comprehensive nature of these scales allows the questionnaire to effectively capture a wide range of food skills, making it a reliable instrument for evaluating basic to intermediate food skills.

#### 3.2.9. Escala de Habilidades Culinárias Domésticas da Atenção Primária à Saúde (EHAPS)—Primary Health Care Home Cooking Skills Scale

This instrument comprehensively assesses cooking skills among primary healthcare workers, covering various aspects of meal planning, preparation, creativity, multitasking, sensory perception, and confidence. The structured response options facilitate data collection and analysis, enabling researchers to gain insights into the cooking abilities and perceptions of healthcare professionals in the primary care setting. It also assesses confidence in preparing food, cooking meat, or using a pressure cooker.

The questionnaire is structured into five theoretical domains with 29 items after a thorough validation (Table 2). The first domain, Planning of Purchases and Meal Preparation, includes six items that assess tasks such as making a shopping list before going to the supermarket, organizing meals for the week, and freezing prepared meals in portions. The second domain, Culinary Creativity, contains five items that evaluate the respondent’s ability to create different dishes from the same ingredients, use leftovers creatively, and adapt recipes based on available ingredients.

The third domain, Preparation Skills and Multitasking, is the most extensive, comprising 12 items. These items cover various food preparation techniques, such as blanching broccoli, chopping onions, and preparing homemade sauces. The fourth domain, Sensory Perception, includes seven items that measure the respondent’s ability to use their senses—taste, smell, and sight—to assess the quality and readiness of food, such as judging the doneness of grilled meat by touch.

Finally, the fifth domain, Confidence, contains four items that assess the respondent’s confidence in performing more advanced culinary tasks, including using a pressure cooker, preparing homemade bread, and adjusting recipes. Each item in the EHAPS is evaluated through a five-point Likert scale, ranging from “Never” (0) to “Always” (4).

The EHAPS is the only instrument that defines a specific range parameter for the responses within the instrument. Each item on the scale generates a score based on the individual’s response (insert the scale here), defining the ranges for Domestic Cooking Skills (DCS) as follows: low DCS ranges from 0 to 29 points (corresponding to ≤25% of the upper score); moderately low DCS ranges from 30 to 58 points (corresponding to >25% and ≤50% of the upper score); moderately high DCS ranges from 59 to 87 points (corresponding to >50% and ≤75% of the upper score); and high DCS ranges from 88 to 116 points (equivalent to >75% of the upper score). This instrument has not been applied in other languages.

The fact that this instrument already presents a score to evaluate the person’s answer is a positive aspect of the standardization and replication of an instrument. However, as it has never been translated into another language, it is difficult to assess its cultural adaptability.

Nevertheless, considering the main objective of this review is to measure actual cooking skills such as preparing a recipe, creating dishes, and using specific kitchen utensils, none of the items in this instrument evaluate these aspects. The main focus is recipe preparation and the individual’s confidence in this ability, without mentioning specific cooking skills.

#### 3.2.10. Cooking Skills Index (CSI)

The questionnaire consists of ten items designed to measure the respondent’s confidence in their ability to perform particular cooking tasks. The ten items in the CSI cover a range of cooking skills, including tasks like stewing food, oven-baking or roasting and others that are available in Table 2.

The scale used for each item is a four-point Likert scale, with the following response options: 0—Not confident; 1—Little confident; 2—Confident; 3—Very confident. The sum of these values generates a score ranging from 1 to 30, which is then proportionally converted to a value out of 100. The closer the score is to 100, the higher the individual’s skill is considered to be. However, no parameter is provided to define the ranges for low, medium, or high cooking skills.

A higher score indicates greater confidence in performing these essential cooking skills. The CSI was developed to provide a reliable instrument for assessing confidence in cooking skills within the Brazilian context but was never translated or adapted to any other culture.

As a short and straightforward instrument, it is easier to use the questions when considering response adherence, which could facilitate translation. However, the items include questions about specific recipes, such as preparing soups, beans, and tomato sauce, which may not be familiar in other cultures, making replication in different contexts more challenging.

Moreover, this instrument does not specifically assess the individual’s skills and is more related to the perception of confidence in preparing recipes or adapting certain preparations with what is available at home.

## 4. Discussion

In the context of the need to modify population habits towards health based on lifestyle improvements that encompass the acquisition and maintenance of an adequate and healthy diet, it is widely recognized that one of the priority areas of research and emerging issues is the need to obtain more data on individuals’ cooking skills across all age groups [1,58]. There is a strong correlation between cooking skills and reduction in the risk of non-communicable diseases like obesity, type 2 diabetes, and cardiovascular diseases [28].

Measuring these skills makes it possible to identify risk groups and target preventive efforts more effectively. Promoting cooking skills can reduce dependence on processed foods and ready meals, leading to a decrease in healthcare costs in the long term. In this way, cooking skills are fundamental to creating public policies that promote food and nutrition education, helping to improve the diet, nutrition, and health of the population [59].

Cooking skills assessment should be performed accurately to determine how various communities engage in the process, influencing their options in relation to food and, ultimately, their well-being [43,60]. In this review, we observed many instruments available in the world literature (n = 12) in various countries, with Brazil studying over half of these instruments.

Most of the studies were conducted in Brazil (n = 17; 45%), and 12 of them were conducted by the same research group using the translated and validated version of the Food and Cooking Skills Questionnaire in Brazilian Portuguese. The research group that widely used this questionnaire in Brazil explains the large number of studies performed in this country. Different from other countries, in Brazil, despite the increase in eating out, people usually prefer to consume homemade meals (requiring cooking skills of their own or of a person who prepares meals at home) or search for restaurants that offer meals similar to homemade (requiring cooking skills from food handlers) [61].

The reduced number of countries where studies with validated instruments were performed to evaluate cooking skills highlights the importance of performing studies in different countries and the need to standardize the instrument to be used in different languages, allowing comparison among various populations. In addition, validating translated versions of an instrument is essential to ensuring the accuracy and relevance of collected data, particularly in epidemiological studies [62].

To guarantee the instrument’s applicability, it is essential to directly assess cooking skills, focusing on specific cooking techniques and competencies, such as boiling, sautéing, and following recipes. These instruments include measurements of confidence or attitudes towards cooking, such as the Food and Cooking Skills Questionnaire, which evaluates confidence in using various cooking techniques and following recipes in some of its domains, and the Cooking and Food Skill Confidence Questionnaire, which assesses self-perceived proficiency in tasks like chopping, mixing, and cooking. The results of our study indicate that the Cooking and Food Skill Confidence Questionnaire appears to be simple in use, presenting no significant interpretive challenges for cooking evaluation.

Instruments that address various issues, including food availability or attitudes towards food, can become lengthy and tiring for respondents, as well as detract from the objective of assessing specific cooking skills. In this way, concise and objective instruments, such as Lavelle et al.’s questionnaire on confidence in cooking skills, prove to be a promising option for standardization and application in various contexts.

In addition, the use of specific and traditional recipes in questionnaires, such as “Can you prepare miso soup?” (cultural adaptation in Japan), may limit the instrument’s applicability to other cultures where these cooking practices are not common. Including questions such as “How confident are you in your ability to cook from basic ingredients?” or “Do you know how to use the kitchen equipment you have?” may distract from the main objective of assessing specific technical skills.

To ensure the robustness and applicability of a cooking skills assessment instrument, it is essential to include well-structured scales that accurately measure participants’ abilities.

For instance, the Food and Cooking Skills Questionnaire uses a combination of Likert scales and multiple-choice questions, effectively capturing nuances in respondents’ attitudes and cooking skills. The Cooking and Food Skill Confidence Questionnaire also employs Likert scales, which effectively measure confidence in specific cooking tasks, providing a detailed view of individuals’ self-perception regarding their kitchen skills.

The inclusion of well-developed scales not only strengthens the internal validity of the instrument but also enhances its ability to discriminate between different levels of cooking skills, allowing for a more in-depth analysis of the collected data. Scales like these offer consistency and reliability, which are crucial for the replicability and generalization of results across different cultural contexts [63,64].

### Study Strengths and Limitations

The rigorous methodology, following a systematic approach and using PRISMA-ScR adapted guidelines, ensures the quality and reliability of the collected and analyzed data. Furthermore, the study identified these instruments and compared their characteristics, strengths, and limitations, providing a clear perspective on the most suitable questionnaires for different research contexts and objectives. A potential limitation of this study is that there may be many high-quality studies on cooking skills that were excluded because they did not undergo proper validation, which was one of the exclusion criteria of this article. However, the importance of this validation process was highlighted during this research.

## 5. Conclusions

This review evaluated 11 validated instruments for assessing cooking skills in adults, focusing on their scale structures, item quantity, response options, and cultural adaptability. A diversity was found in the strategies of these instruments to measure specific cooking techniques, confidence, and attitudes toward cooking. Some instruments offered extensive assessments with numerous items across multiple domains, providing a thorough evaluation but posing challenges regarding respondent burden and cultural adaptation. Others were more concise, allowing for easier administration and replication across different cultural settings, though potentially lacking depth in capturing complex cooking skills.

Well-structured scales with clearly defined domains and reliable response options were considered essential criteria for strengthening the instrument. Instruments that utilized the direct cooking technique in their composition, without country-specific preparations, were more effective in cross-cultural replications, ensuring more translations to different languages. Selecting instruments that balance comprehensiveness with practicality is crucial, especially in global studies where cultural differences are significant. Accurate assessment of cooking skills is essential for guiding public health policies and interventions, as these skills are strongly linked to healthier dietary behaviors and reduced reliance on processed foods. This can contribute to decreasing non-communicable diseases and associated healthcare costs, highlighting the importance of carefully choosing thorough and culturally adaptable instruments.

By exploring the various instruments used to assess cooking skills in adults, this review highlights the “Cooking and Food Skill Confidence Questionnaire” as one of the most appropriate instruments for evaluating cooking skills since it has been translated and validated for various cultures and has undergone rigorous validation processes, including factor analyses and internal consistency tests, showing high Cronbach’s alpha values, which indicate the reliability and consistency of the measures over time. It covers a wide range of cooking skills, from basic food preparation techniques to meal planning, in a direct and specific manner, and it has a design that minimizes respondent fatigue.

It is important to highlight that to measure cooking skills effectively, it is crucial to go beyond behaviors like meal preparation frequency and focus on practical techniques that form the core of culinary expertise. While validated instruments show cross-cultural applicability, they often emphasize self-perception, attitudes, and routines, overlooking essential skills like chopping, baking, or frying. Future research should explore the relationship between these technical abilities and cultural heritage passed down through generations, as such ancestral knowledge can significantly shape an individual’s cooking skills and practices. Integrating these dimensions could provide more comprehensive and culturally meaningful assessments.

## Figures and Tables

**Table 1:** Data extraction table for studies that published a validated instrument of cooking skills.

Reference	Authors Name and Year	Study Origin	Aim	Translation/ Development and Validation/Application	Version of the Instrument
Food and Cooking Skills Questionnaire					
[21]	WRIEDEN et al., 2007	UK	Examine the impact of a community-based food skills intervention on cooking confidence, food preparation methods, and dietary choices through an exploratory trial	Development and validation	Original
[22]	MICHAUD, 2007	USA	This study aims to detail the development and evaluation of instruments to measure the effectiveness of a nutrition education program in cooking skills	Application	Original
[23]	CONDRASKY et al., 2011	USA	Development of psychosocial scales for evaluating the impact of a culinary nutrition education program on cooking and healthful eating	Development and validation	Original
[24]	BERNARDO et al., 2021	Brazil	Association of personal characteristics and cooking skills with vegetable consumption frequency among university students	Application	Brazillian translation
[25]	JOMORI et al., 2017	Brazil	Construction and validity of Brazilian cooking skills and healthy eating questionnaire	Development, validation, and application	Brazillian translation
[26]	BERNARDO et al., 2018	Brazil	Positive impact of a cooking skills intervention among Brazilian university students	Application	Brazillian translation
[27]	LOPES, 2019	Brazil	Evaluation of the cooking skills and healthy eating habits of nutrition students at the federal university of Rio Grande do Sul	Application	Brazillian translation
[28]	ELPO, 2020	Brazil	Evaluation of the impact of a culinary intervention on the culinary skills of individuals with type 2 diabetes mellitus	Application	Brazillian translation
[29]	DE BORBA et al., 2020	Brazil	To explore individuals’ self-efficacy in cooking and consuming fruits and vegetables, examining their confidence and the factors that influence it	Application	Brazillian translation
[30]	BERNARDO et al., 2017	Brazil	Describe the study protocol and evaluation framework for the Nutrition and Culinary in the Kitchen program. This program, based on the Cooking with a Chef program in the United States, aims to develop cooking skills in university students	Application	Brazillian translation
[14]	JOMORI et al., 2021	Brazil	Describe the cross-cultural adaptation process of the questionnaire to evaluate Brazilian university students’ cooking skills and healthy eating, providing some lessons for nutrition research	Application	Brazillian translation
[31]	DEZANETTI et al., 2022	Brazil	To estimate the likelihood of meal preparation and where university students consumed meals before and during the COVID-19 pandemic based on their characteristics and cooking skills	Application	Brazillian translation
[32]	DE SOUZA; KOTZIAS, 2022	Brazil	Characterize the cooking skills and dietary practices of Nutrition students at a public university in Brazil	Application	Brazillian translation
[33]	PELONHA, 2023	Brazil	Evaluate the cooking skills of university students and their association with overweight and obesity	Application	Brazillian translation
[34]	SOARES, 2023	Brazil	Evaluate the impact of cooking skills and COVID-19 on culinary consumption, attitudes, and behaviors	Application	Brazillian translation
[4]	LILLQUIST et al., 2022	USA	Comparing nutrition knowledge, eating habits, cooking skills, and weight in patients before and after an eight-week culinary and nutrition intervention	Application	Original
Short questionnaire for assessing the impact of cooking skills interventions					
[35]	BARTON; WRIEDEN; ANDERSON, 2011	UK	Validity and reliability of a short questionnaire for assessing the impact of cooking skills interventions	Development, validation, and application	Original
Cooking Skills Scale					
[36]	HARTMANN; DOHLE; SIEGRIST, 2013	Switzerland	Importance of cooking skills for balanced food choices	Validation and application	Original
[37]	KOWALKOWSKA; POÍNHOS; RODRIGUES, 2018	Portugal	Translation of a cooking skills instrument and its comparison to the sociodemographic characteristics of the Portuguese population	Translation, validation, and application	
Canadian Community Health Survey (CCHS) Rapid Response Annex on Food Skills					
[38]	SLATER; MUDRYJ, 2016	Canada	Compare food habits and cooking skills of Canadians	Application	Original
[39]	FERNANDEZ et al., 2019	Canada	Compare if there is the relation between full-time employment, diet quality, and food skills of Canadian parents	Application	Original
Cooking and Food Skill Confidence Questionnaire					
[11]	LAVELLE et al., 2017	Ireland	Development and validation of measures to assess cooking skills and food skills	Development and validation	Original
[40]	LAVELLE et al., 2016	UK	Explore how cooking skills develop and vary across different age groups, providing insights into the relationship between age and culinary proficiency	Application	Original
[41]	COSTA et al., 2023	Portugal	To validate the questionnaire to assess the food and cooking skills of the young Portuguese adult population through the assessment of psychometric properties	Translation, validation, and application	Portuguese translation
[42]	COMERT; OZBEK, 2023	Turkey	Translation to Turkish and validation of the Turkish version of the cooking and food skills confidence questionnaire	Translation and validation	Turkish translation
[43]	ARSLAN et al., 2022	Turkey	Determining food preparation and cooking skills, eating behaviors, and evaluating the relationship between these skills and eating behaviors in individuals with overweight or obesity	Application	Turkish translation
[44]	DOS SANTOS; SPRADA; VILELA, 2024	Brazil	To perform the translation and transcultural adaptation of the Cooking Skills questionnaire to make this instrument available to healthcare professionals, especially nutritionists, in future research	Translation, validation, and application	Brazilian translation
Cooking Skills Index					
[45]	MARTINS et al., 2019b	Brazil	Cooking Skills Index: Development and reliability assessment	Development and validation	Original
[15]	MARTINS et al., 2019a	Brazil	Examine how parents’ cooking skills influence the dietary habits of school-aged children	Application	Original
[46]	MARTINS et al., 2020	Brazil	To identify if there is any relation between parents’ cooking skills confidence in reducing children’s consumption of ultra-processed foods	Application	Original
Cooking and Food Provisioning Action Scale (CAFPAS)					
[47]	LAHNE; WOLFSON; TRUBEK, 2017	USA	Development of the Cooking and Food Provisioning Action Scale (CAFPAS)	Development and validation	Original
[48]	KIM; CHOI, 2023	South Korea	To investigate the relationship between cooking practices and healthy eating habits among young Korean adults in their twenties	Translation, validation, and application	
Scale to Measure Cooking Skills					
[49]	BECH-LARSEN; TSALIS, 2018	Denmark	Development of scales to measure cooking skills and other competences	Development and validation	Original
Food Skills Questionnaire (FSQ)					
[50]	KENNEDY et al., 2019	Canada	Validity and Reliability of a Food Skills Questionnaire	Validation and application	Original
[51]	MAHMOUD et al., 2021	Canada	Using the Food Skills Questionnaire (FSQ) to evaluate a cooking intervention for university students: a pilot study	Application	Original
[52]	MORGAN; MATTHEWS, 2023	Canada	Exploring young women’s perceptions of their food skills	Application	Original
The Japan Gerontological Evaluation Study (JAGES)					
[53]	TANI; FUJIWARA; KONDO, 2020	Japan	Cooking skills related to potential benefits for dietary behaviors and weight status among older Japanese men and women: A cross-sectional study from the JAGES	Translation, validation, and application	Original
Escala de habilidades culinárias domésticas da atenção primária à saúde (ehaps)					
[54]	TEIXEIRA et al., 2022	Brazil	Development of an instrument to measure domestic cooking skills in primary healthcare	Development and validation	Original

**Table 2:** Description of domains and items of each instrument identified in this review.

DOMAINS AND THEIR ITEMS
Food and Cooking Skills Questionnaire (8 domains—63 items)
**I. Availability and Accessibility of Fruits and Vegetables Index (2-point scale: yes or no)**: 1. Did you have pure (100%) fruit juice in your home last week?; 2. Did you have fresh fruit in your home last week?; 3. Did you have raw or cooked vegetables in your home last week?; 4. Did you have salad in your home last week?; 5. In the last week, were fruit and vegetables on the kitchen counter or somewhere in the open?; 6. In the last week, was 100% fruit juice or cut up fresh fruit on the front shelf of the refrigerator as a snack?; 7. In the last week, were cut up fresh vegetables on the front shelf of the refrigerator as a snack?; 8. In the last week, were vegetables in the refrigerator prepared so they readily could be used in a meal? **II. Cooking Attitude Scale (5-point scale: Strongly disagree to Strongly agree)**: 9. I do NOT like to cook because it takes too much time. *; 10. Preparing meals at home would NOT improve the health of my diet. *; 11. Cooking meals is a good use of my time.; 12. I enjoy cooking.; 13. It is important to know how to prepare food.; 14. Cooking is fun.; 15. I do NOT like to prepare meals at home because it costs too much money. *; 16. It is NOT important that I know how to cook. *; 17. Cooking is interesting.; 18. Meals made at home are affordable.; 19. It is important to eat the recommended 2 cups of fruit each day.; 20. It is important to eat the recommended 2 ½ cups of vegetables each day.; 21; It is easy to prepare meals.; 22. Cooking is frustrating. *; 23. I like trying new recipes.; 24. It is too much work to cook. *; 25. Making meals at home helps me to eat more healthfully.; 26. I find cooking tiring. * **III. Cooking Behavior Scale (5-point scale: Not at all, 1 to 2 times this month, once a week, several times each week, about every day)**—During the past month, how often did you do the following?; 27. Prepare meals from basic ingredients (such as whole fresh produce, raw chicken, etc.).; 28. Prepare meals using convenience items (such as bagged salad, prepared mashed potatoes, pre-shredded carrots, deli rotisserie chicken).; 29. Reheat or use leftovers in another meal. **IV. Produce Consumption Self-Efficacy Scale (5-point scale: Not at all confident to Extremely confident)**: 30. Eat fruits and vegetables at every meal, every day.; 31. Eat fruits or vegetables as a snack, even if everybody else were eating other snacks.; 32. Eat the recommended 9 half-cup servings of fruits and vegetables each day. **V. Cooking Self-Efficacy Scale (5-point scale: Not at all confident to Extremely confident)**: 33. Cook from basic ingredients (e.g., whole lettuce heads, fresh tomatoes, raw chicken).; 34. Follow a written recipe (e.g., preparing fresh salsa from tomatoes, onion, garlic, jalapeno peppers).; 35. Prepare dinner from items you currently have in your pantry and refrigerator.; 36. Use knife skills in the kitchen.; 37. Plan nutritious meals.; 38. Use basic cooking techniques.; 39. Reuse leftovers for another meal.; **VI. Self-Efficacy for Using Basic Cooking Techniques Scale (5-point scale: Not at all confident to Extremely confident)**: 40. Boiling; 41. Simmering; 42. Steaming; 43. Deep frying; 44. Sautéing; 45. Stir-frying; 46. Grilling; 47. Poaching; 48. Baking; 49. Roasting; 50. Stewing; 51. Microwaving. **VII. Self-Efficacy for Using Fruits, Vegetables, and Seasonings Scale (5-point scale: Not at all confident to Extremely confident)**: 52. Fresh or frozen green vegetables (e.g., broccoli, spinach); 53. Root vegetables (e.g., potatoes, beets, sweet potatoes); 54. Fruit (e.g., peaches, watermelon); 55. Herbs and spices (e.g., basil, thyme, cayenne pepper) **VIII. Knowledge of Cooking Terms and Techniques Evaluation (multiple-choice questions)**: 56. Cooking peaches briefly in boiling water then cooling in ice water to remove the skins is an example of:—Blanching;—Poaching;—Broiling;—Don’t know; 57. If a recipe tells you to sauté an onion, you should cook it:—In a basket set above boiling water.;—In a pan with a small amount of hot oil.;—In a pan with a small amount of water.;—Don’t know.; 58. A diced potato should be cut into:—Long, thin matchstick-size pieces.;—Very small and uneven pieces.;—Cubes usually ¼ to ¾ inch in size.;—Don’t know.; 59. Water is simmering when:—Steam begins to form.;—Tiny bubbles collect on the bottom and sides of the pan.;—Bubbles rise rapidly and break on the surface.;—Don’t know.; 60. Sweet potatoes are roasting when they are:—Cooked by dry heat in a hot oven.;—Cooked in a hot oven with liquid in the pan.;—Cooked in a covered pan with a small amount of liquid.;—Don’t know.; 61. What is the term for preparing all ingredients, gathering equipment, and organizing your work area before beginning to cook?—Production stage;—Blanching;—Mise en place;—Don’t know; Based on the following recipe, respondents should answer the next questions: Orange Smoothie 1 cup fat-free vanilla yogurt ½ cup sweet potatoes, cooked, cooled, and mashed 1 cup orange juice ½ tsp vanilla extract 1 cup ice In a blender, crush ice. Add remaining ingredients and blend on high until smooth. Serve immediately. Yield: 2 smoothies. 62.To accurately measure 1 cup of orange juice for this recipe:—Set a liquid measuring cup on a level surface, bend down and pour in the juice to the desired level.;—Hold a dry measuring cup at eye level and pour in juice from another container to the desired level.;—Set a dry measuring cup on a level surface, bend down and pour the juice to the desired level.—Don’t know. 63. Which is best for measuring the vanilla extract in this recipe?—Image of standard measuring spoons;—Image of a liquid measuring cup;—Image of a tablespoon;—Don’t know. * These items should be reverse scored.
Short Questionnaire for Assessing the Impact of Cooking Skills Interventions (5 domains—19 items)
1. What kind of cooking do you do at the moment? (please tick as many boxes as appropriate) 1.1 Cook convenience foods and ready-meals; 1.2. Put together ready-made ingredients to make a complete meal; 1.3. Prepare dishes from basic ingredients; 2. How often do you prepare and cook a main meal from basic ingredients?; 3 How confident do you feel about being able to cook from basic ingredients?; 4. How confident do you feel about following a simple recipe?; 5. How confident do you feel about tasting foods that you have not eaten before?; 6. How confident do you feel about preparing and cooking new foods and recipes?; (**from 2 to 6 option of response is a likert scale of 7 points from “Extremely Confident” to “Not at all Confident”**); 7. How often do you eat fruit?; 8. How often do you eat vegetables or salad?; 9. How often do you eat pasta or rice?; 10. How often do you eat baked, boiled or mashed potatoes?; 11. How often do you eat chips, fried or roast potatoes?; 12. How often do you eat fish or fish products?; (**from 7 to 12 option of response is a likert scale of 7 points with those options: never; less than once a week; once a week; 2–4 times a week; 5–6 times a week; once a day and more than once a day**) 13. Do you think you will increase the amount of fruit and vegetables you eat in the next 6–12 months? (**responses in a 5 points scale from “definitely not” to “definitely” and an extra option with “Don’t know”**); 14. How many portions of fruit and vegetables do you think health experts recommend eating every day? (**from none to five or more on a 6 points scale and an extra option with “Don’t know”**); 15. How many portions of fruits or vegetables does each of the following provide? (please tick one box per line)—each line has options from zero to three and a “don’t know” option; 15.1 Portions of fruit or vegetables in a medium glass of unsweetened orange juice; 15.2. Portions of fruit or vegetables in one glass of orange squash; 15.3. Portions of fruit or vegetables in a thin slice of tomato; 15.4. Portions of fruit or vegetables in three heaped tablespoons of carrots; 15.5. Portions of fruit or vegetables in one medium apple; 15.6. Portions of fruit or vegetables in one small raspberry yogurt; 16. Do you eat food past its ‘use by’ date?; 17. Do you follow the instructions for storage on packaged foods?; 18. Do you check that food is piping hot when re-heating?; 19. Do you wash fruit and vegetables that don’t need to be peeled before eating?; (**from 16 to 19, the options are on a scale from always to never and a “Don’t know” option**)
Cooking Skills Sscale (CSS) (7 items)
**I. The items were rated on a six-point Likert scale ranging from 1 = do not agree at all to 6 = totally agree**. 1. I consider my cooking skills as sufficient; 2. I am able to prepare a hot meal without a recipe; 3. I am able to prepare gratin; 4. I am able to prepare soup; 5. I am able to prepare sauce; 6. I am able to bake a cake; 7. I am able to bake bread.
Cooking Skills Scale Sdaptation—The Japan Gerontological Evaluation Study (JAGES) (7 items)
**I. The items were rated on a six-point Likert scale ranging from 1 = do not agree at all to 6 = totally agree**. 1. How do you assess your overall cooking skills?; 2. Can you peel fruits and vegetables?; 3. Can you boil eggs and vegetables?; 4. Can you grill fish?; 5. Can you make stir-fried meat and vegetables?; 6. Can you make miso soup? 7. Can you make stewed dishes?
The Canadian Community Health Survey (CCHS) Rapid Response Annex on Food Skills (4 domains—18 items)
**I. self-perceived eating habits**: 1. In general, would you say that your eating habits are (**response from 1. excellent to 5. poor); II. meal preparation habits**: 2. How would you describe your ability to cook from basic ingredients? (this item has response options that the interviewer must read to the participant to choose one of those: 2.1. I don’t know where to start when it comes to cooking; 2.2. I can do things such as boil an egg or cook a grilled cheese sandwich, but nothing more advanced; 2.3. I can prepare simple meals but nothing too complicated; 2.4. I can cook most dishes if I have a recipe to follow; 2.5. I can prepare most dishes; 2.6. I frequently prepare sophisticated dishes); **III. mechanical skills and food**: 3. How would you rate your skills (**responses on a scale from 1 very good to 5. Very limited/no skills**) 3.1. in using a kitchen knife safely?; 3.2. in peeling, chopping, or slicing vegetables or fruit?; 3.3. in freezing vegetables or fruit, from raw to bagged in your home freezer?; 3.4. in canning from raw ingredients to finished products in sealed glass jars?; 3.5. in cooking a piece of raw meat/chicken/fish?; 3.6. in cooking a soup, stew, or casserole from scratch?; 3.7. in baking muffins or cake using a pre-packaged mix?; 3.8. in baking muffins or cake from scratch with a recipe?; **IV. Healthy habits**: 4. Have you ever adjusted a recipe to make it healthier?; 5. How did you make it healthier? (**here the participant must check all options that apply from various provided options**); 6. When the season permits, do you grow vegetables, herbs, or fruits at home or in a community garden? (**from 4 to 6 respond with YES or NO**).
Cooking and Food Skill Confidence Questionnaire (2 domains—32 items)
**I. Cooking Skills Confidence Measure**: how good you are at each skill, on a **scale of 1–7, where 1 is very poor and 7 is very good**: 1. Chop, mix, and stir foods; 2. Blend foods to make them smooth, like soups or sauces; 3. Steam food; 4. Boil or simmer food; 5. Stew food; 6. Roast food; 7. Fry/stir-fry food in a frying pan/wok with oil or fat; 8. Microwave food; 9. Bake goods; 10. Peel and chop vegetables; 11. Prepare and cook raw meat/poultry; 12. Prepare and cook raw fish; 13. Make sauces and gravy from scratch; 14. Use herbs and spices.; **II. Food Skills Confidence Measure**: How good you are at each skill, on **a scale of 1–7**, **where 1 is very poor and 7 is very good**: 15. Plan meals ahead; 16. Prepare meals in advance; 17. Follow recipes when cooking; 18. Shop with a grocery list; 19. Shop with specific meals in mind; 20. Plan how much food to buy; 21. Compare prices before buying food; 22. Know what budget you have to spend on food; 23. Buy food in season to save money; 24. Buy cheaper cuts of meat to save money; 25. Cook more or double recipes that can be used for another meal; 26. Prepare or cook a healthy meal with only a few ingredients on hand; 27. Prepare or cook a meal with limited time; 28. Use leftovers to create another meal; 29. Keep basic items in your cupboard for putting meals together; 30. Read the best-before date on food; 31. Read the storage and use-by information on food packets; 32. Read the nutrition information on food labels; 33. Balance meals based on nutrition advice on what is healthy.
Cooking Skills Index (10 items)
**I. The items were rated on a four-point scale: (0) not confident, (1) little confident, (2) confident, and (3) very confident**. 1. Stewing a food; 2. Oven-baking/Roasting; 3. Seasoning meat using only natural seasonings; 4. Following a simple recipe; 5. Making a homemade tomato sauce using only tomatoes and natural seasonings; 6. Preparing a homemade soup; 7. Cooking beans in a pressure cooker; 8. Grilling meat; 9. Preparing a simple homemade cake; 10. Preparing a lunch or dinner by combining foods and spices already available in the house without a recipe.
Cooking and Food Provisioning Action Scale (CAFPAS) (3 domains—28 items)
**All items were presented with 7-point Likert scales, with response options from “Strongly Disagree” (coded as “1”) to “Strongly Agree” (coded as “7”).** **I. food self-efficacy (13 items)** 1. I feel limited by my lack of cooking knowledge; 2. I can always manage to decide what I would like to eat at any given time; 3. When preparing food, I am confident that I can deal with unexpected results; 4.When preparing food, it is easy for me to accomplish my desired results; 5. In preparing food, I can solve most problems with enough effort; 6. I am comfortable preparing food; 7. I know how to use the kitchen equipment I have; 8. I am involved in daily meal preparation; 9. When I shop for food, I know how I will use the ingredients I am purchasing; 10. I am confident creating meals from the ingredients I have on hand; 11. Before I start cooking, I usually have a mental plan of all the steps I will need to complete; 12. When presented with two similar products to purchase, I feel confident choosing between them; 13. I know where to find the ingredients I need to prepare a meal; **II. attitude (10 items)**: 14. I find cooking a very fulfilling activity; 15. For me, cooking is just something to get through as quickly as possible; 16. Compared to other activities, cooking brings me little enjoyment; 17. If I try making a new type of food and it does not come out right, I usually do not try to make it again; 18. I think a lot about what I will cook or eat; 19. I prefer to spend my time on more important things than food; 20. If everything else is equal, I choose to cook rather than have food; prepared by someone else; 21. I feel like cooking is a waste of effort; 22. I am inspired to cook for other people, like my family or friends; 23. I feel burdened by having to cook for other people, like my family or friends; **III. structure (5 items)**: 24. I wish that I had more time to plan meals; 25. I have a hard time finding enough time to prepare the food I’d like to eat; 26.My family responsibilities prevent me from having time to prepare meals; 27. My social responsibilities prevent me from having the time to prepare meals; 28. My job responsibilities prevent me from having the time to prepare meals.
Scale to Measure Cooking Skills (2 domains—26 items)
**I. Respondents indicated yes/no to a stem that began, “Did you, within the previous year, prepare ….”—Experience** “Did you, within the previous year, prepare …” 1. Carrot slaw; 2. Omelet; 3.; Sauce béarnaise; 4. Mussels (steamed); 5. Mayonnaise; 6. Steak (fried); 7. Lamb stew; 8. Fish soup; 9. Sushi; 10Bread or buns; 11. Pancakes; 12. Marzipan ring cake; 13. Cream puffs; 14. Fragilité; 15. Eggs (boiled); 16. Garlic (chopped); 17. Meat stuffing (mixed); 18. Egg whites (whipped); 19. Soup (blended); 20. Chicken (carved); 21. Fish (filleted); **II. Respondents indicated true/false in response to a stem that began, “Are the following statements…?”—Knowledge** “Are the following statements…?” 1. Fruits and vegetables should be rinsed; 2. Salmonella is a fat that is found in salmon; 3. One should wash one’s hands before preparing food; 4. Food passed the best before date should not be eaten; 5. Frozen rye bread retains its quality for one year.
Food Skills Questionnaire (FSQ) (3 domains—39 items)
**Most questions used an 11-point scale (0−100); however, attitudinal questions were on a 6-point scale of strongly disagree to strongly agree and food safety questions were primarily on a 5-point scale of never to always. I—Food Selection and Planning**—1. How often do you check best-before dates?; Rate your confidence in: 2. Budgeting for groceries; 3. Meal planning; 4. Selecting vegetables and fruits; 5. Reading food labels; 6. Planning a meal using only foods already in your home; 7. Adjusting a recipe to make it healthier. What percentage of the time do you: 8. Use a grocery list?; 9. Buy a variety of vegetables?; **II—Food Preparation**—How many times per week do you make the following home-prepared meals? 10. Breakfast; 11. Lunch; 12. Dinner; 13. What percentage of the meals you prepare are balanced?; 14. Describe your ability to prepare meals. To what extent do you agree or disagree: 15. Food preparation takes too much time*; 16. Food preparation is too much work; 17. Making meals at home helps me to eat healthier; 18. Food preparation is enjoyable. Rate your confidence in: 19. Using knives in the kitchen; 20. Peeling, chopping, and slicing vegetables and fruits; 21. Using vegetables in food preparation; 22. Using beans and lentils in food preparation; 23.Preparing food from basic ingredients; 24. Following a simple recipe;25. Boiling, steaming, or stewing; 26. Stir-frying or pan-frying; 27. Baking, grilling, or roasting; 28. Choosing a spice or herb; 29. Preparing new foods or recipes. **III—Food Safety and Storage**—How often do you: 30. Wash countertops before preparing food?; 31. Wash your hands before preparing food?; 32. Use microwave/fridge/cold water to thaw frozen meat?; 33. Separate raw meat, poultry, and seafood?; 34. Cook foods to the correct internal temperature?; 35. Wash fruits and vegetables?; 36. Put leftovers in the refrigerator within 2 h?; 37. Follow instructions for storage on packaged foods?; 38. Check that food is heated throughout when reheating?; Rate your confidence in: 39. Staying safe in the kitchen (e.g., avoiding burns and cuts). * Question was reverse-coded.
Escala de Habilidades Culinárias Domésticas da Atenção Primária à Saúde (EHAPS)—Primary Health Care Home Cooking Skills Scale (5 domains—29 items) *
**Response options on a five-point Likert scale (0 = strongly disagree and 5 = strongly agree).** **(I) Shopping Planning and Meal Preparation**: 1. Plan the menu considering the use of unconventional parts of food; 2. Prepare broths from fresh ingredients; 3. Determine the quantity of food to be purchased based on the number of people eating at home; 4. Check the items I have at home before buying food. **(II) Culinary Creativity**: 5. Prepare different culinary recipes using the same ingredients; 6. Create different sauces to vary meals; 7. Use leftovers to prepare a new culinary recipe; 8. Handle unexpected situations when cooking; 9. Adapt culinary recipes with the ingredients I have at home; 10. Correct the acidity of sauces using fresh ingredients, such as carrot; 11. Prepare homemade tomato sauce. **(III) Preparation and Multitasking Skills**: 12. Do other household tasks; 13. Handle a pending issue over the phone while the pots are on the stove; 14. Prepare lunch/dinner from scratch in less than 30 min. **(IV) Sensory Perception**: 15. Adjust the amount of culinary seasonings while tasting food during preparation; 16. Combine foods based on their flavor; 17. Identify the cooking point of foods according to their consistency; 18. Recognize when a white sauce is ready based on its thick texture; 19. Identify if foods are suitable for consumption based on their sensory characteristics. **(V) Confidence**: 20. Cook tough meats, like muscle, in liquid to make them tender; 21. Use the pressure cooker by myself; 22. Prepare a sugar syrup; 23. Follow a culinary recipe from start to finish; 24. Prepare homemade bread; 25. Cook foods according to the point indicated in the culinary recipe; 26. Roast a whole bird; 27. Adjust the quantity of ingredients in a culinary recipe for more people; 28. Convert universal measurements; 29. Prepare a simple cake without instructions. * This instrument was originally published in Brazilian Portuguese and has been freely translated into English for the purposes of this study

The **bold** text in the table refers to the domains present in each instrument.

## Data Availability

No new data were created or analyzed in this study. Data sharing is not applicable to this article.
